# Genetic diagnosis of Beckwith Wiedemann syndrome and Silver-Russell syndrome

**DOI:** 10.1186/1687-9856-2013-S1-O44

**Published:** 2013-10-03

**Authors:** Yoo-Mi Kim, Gu-Hwan Kim, Beom Hee Lee, Jin-Joo Lee, Seong-Hoon Choi, Ju Yeon Lee, Han-Wook Yoo

**Affiliations:** 1 Medical Genetics Center, University of Ulsan College of Medicine, Seoul, Korea; 2 Department of Pediatrics, Asan Medical Center, University of Ulsan College of Medicine, Seoul, Korea

## 

Beckwith-Wiedemann syndrome (BWS) is fetal overgrowth syndrome, whereas Silver-Russell syndrome (SRS) is characterized by pre- or postnatal growth retardation. BWS and SRS share molecular epigenetic defects in chromosome 11p15, where two imprinting centers, *LIT1*-differentially methylated region (MDR) and *H19*-DMR, exist. A small number of patients with SRS harbor maternal uniparental disomy of chromosome 7q as well. Extensive genetic analyses including methylation specific (MS) PCR-RFLP, MS-MLPA, microsatellite markers or MS-pyrosequencing analysis were performed using genomic DNA obtained from peripheral leukocytes to identify the epigenetic detects in patients with BWS and SRS. Ten out of 14 BWS patients (71.4%) showed hypomethylation in *LIT1*-DMR. One BWS patient harbored hypermethylation in *H19*-DMR (7.1%). Two BWS patients had both *H19*-DMR and *LIT1*-DMR defects, one of whom has paternal UPD at chr. 11 (14.3%). Eleven out of 13 SRS patients (78.6 %) showed hypomethylation in *H19*-DMR. One SRS patient (6.7%) had UPD at 7q. With MS-pyrosequencing analysis, epigenetic defects were identified in 93.1% of BWS patients and 85.7% of SRS patients. These positive rates are higher than previously reported positive rates, 80% in BWS and 50% in SRS. In addition, with MS-pyrosequencing analyses, quantification of methylation defects was available, which could identify partial methylation defects that were not revealed by MS-PCR-RFLP or MS-MLPA. The validity of MS-pyrosequencing method for the genetic diagnosis of BWS or SRS is needed to be investigated in a large patient cohort.

